# ALK, NUT, and TRK Do Not Play Relevant Roles in Gastric Cancer—Results of an Immunohistochemical Study in a Large Series

**DOI:** 10.3390/diagnostics12020429

**Published:** 2022-02-07

**Authors:** Marie-Isabelle Glückstein, Sebastian Dintner, Silvia Miller, Dmytro Vlasenko, Gerhard Schenkirsch, Abbas Agaimy, Bruno Märkl, Bianca Grosser

**Affiliations:** 1 General Pathology and Molecular Diagnostics, Medical Faculty, University of Augsburg, 86159 Augsburg, Germany; Marie-Isabelle.Glueckstein@uk-augsburg.de (M.-I.G.); Sebastian.dintner@uk-augsburg.de (S.D.); Silvia.Miller@uk-augsburg.de (S.M.); Bianca.Grosser@uk-augsburg.de (B.G.); 2 General, Visceral, and Transplantation Surgery, Medical Faculty, University of Augsburg, 86159 Augsburg, Germany; Dmytro.Vlasenko@uk-augsburg.de; 3 Tumor Data Management, Medical Faculty, University of Augsburg, 86159 Augsburg, Germany; Gerhard.Schenkirsch@uk-augsburg.de; 4 Institute of Pathology, Friedrich-Alexander-University Erlangen-Nürnberg, University Hospital Erlangen, 91054 Erlangen, Germany; Abbas.Agaimy@uk-erlangen.de

**Keywords:** gastric cancer, TRK, ALK, NUT, immunohistochemistry

## Abstract

ALK, NUT, and TRK are rare molecular aberrations that are pathognomonic for specific rare tumors. In low frequencies, however, they are found in a wide range of other tumor entities. This study aimed to investigate the frequency, association with clinicopathological characteristics, and prognosis of the immunohistochemical expressions of ALK, NUT, and TRK in 477 adenocarcinomas of the stomach and gastroesophageal junction. Seven cases (1.5%) showed an expression of TRK. In NGS, no *NTRK* fusion was confirmed. No case with ALK or NUT expression was detected. *ALK*, *NUT,* and *NTRK* expression does not seem to play an important role in gastric carcinomas.

## 1. Introduction

Gastric cancer is ranked as the sixth most common cancer entity worldwide, having accounted for approximately 780,000 cancer-associated deaths in 2018 [1]. To date, only a few prognostic and therapeutic biomarkers have been identified for gastric cancer. To date, the most important therapeutic marker in gastric carcinoma is HER2 overexpression [2]. In addition, MSI status and high PDL1 expression are independent positive prognostic factors in gastric carcinoma [3,4,5], while aberrant E-cadherin expression is considered an unfavorable prognostic factor and even a negative predictive factor for chemotherapy response [6]. Additionally, two generally accepted molecular classifications for gastric carcinomas have been proposed that have both prognostic and therapeutic implications, namely the Cancer Genome Atlas (TCGA) and Asian Cancer Research Group (ACRG) classification [7,8]. Furthermore, MET amplification and overexpression are thought to play a crucial role in gastric carcinogenesis, but this remains to be found as a predictive factor for the response to anti-MET antibodies [5].

In addition to common drivers, such as *RAS* and *RAF* genes, there exists a group of rare molecular alterations that are mainly associated with specific tumor types but occur at a low frequency in completely different tumor entities as drivers with high oncogenic energy. Currently, few studies evaluating these aberrations in gastric cancer with small sample sizes exist. Anaplastic lymphoma kinase (*ALK*) fusions are known as oncogenic drivers of anaplastic large-cell lymphoma (ALCL), which is ALK positive in 50% of cases [9,10]. In non-small cell lung cancer (NSCLC), *ALK* rearrangements are detected in approximately 5% of cases. In this relatively small proportion of NSCLCs, tyrosine kinase inhibitors represent a valid therapy option [11,12]. To date, ALK alteration has been detected in only a few cases of gastric carcinoma [13,14,15].

Neurotrophic tyrosine receptor kinases (*NTRK*s) consist of three protooncogenes (*NTRK* 1, 2 and 3) encoding the three transmembrane proteins of the tropomyosin receptor kinases receptor family (TRKs) A, B, and C. The TRKs profoundly influence the development and function of the central as well as the peripheral nervous systems. Gene fusion can give rise to a chimeric oncogene represented by the ligand-dependent constitutive, uncontrolled TRK activation and thus uncontrolled activation of underlying signaling pathways. In this way, cell proliferation, migration, synaptic formatting, survival, but also invasion and angiogenesis are promoted. Similarly to *ALK* alterations in ALCL, *NTRK* alterations are specific to infantile fibrosarcoma, congenital mesoblastic nephroma, lipofibromatosis-like and inflammatory myofibroblastic tumor-like neoplasms, and secretory carcinomas of the breast and salivary glands and are potent molecular drivers of disease in these rare tumor entities. However, *NTRK* fusions also occur at a low frequency in a wide variety of entities, which enables the tumor-agnostic use of NTRK inhibitors [16,17,18]. To date, two TRK inhibitors, namely larotrectinib and entrectinib, have been approved for the treatment of solid tumors with *NTRK* fusion. Others are currently being evaluated in clinical trials [17,18,19,20,21]. In 2020, the first case of gastric carcinoma was reported in which both an *NTRK* gene fusion and the transcript of the same could be detected [22].

Nuclear protein in testis (*NUT*) is of clinical relevance in the context of so-called NUT midline carcinomas (NC). These highly aggressive tumors often, but not exclusively, originate from or have contact with the midline structures. *NUT* becomes the molecular driver through gene fusion with certain partner genes [23]. To date, there is only one documented case of NC localized in the stomach, which was reported by Dickson et al. in 2018 [24].

The aim of this retrospective study was to determine whether and to what extent other molecular aberrations that have been previously associated with very specific and rather rare tumors play a role in gastric cancer in a large Western cohort. For this purpose, we evaluated the frequency of alterations in ALK, NUT, and TRK in 477 carcinomas of the stomach and gastroesophageal junction.

## 2. Materials and Methods

### 2.1. Patients

Surgical resection specimens from 511 patients with adenocarcinomas of the stomach and the gastroesophageal junction that were treated between 2005 and 2018 at the Department of Visceral Surgery of the University Hospital Augsburg were included in this study (AEGII and III according to Siewert and Stein [25]). Tumors from 34 patients were excluded from this study, because of the low tumor percentage on the TMA and the final cohort consisted of 477 tumors. Among the tumors, 347 were treated with surgery alone, and 130 patients received neoadjuvant chemotherapy. Detailed clinical characteristics are summarized in Table 1.

Response to preoperative chemotherapy was histopathologically determined and was classified into three tumor regression grades (TRG): TRG1b, TRG2, TRG3, corresponding to <10%, 10%–50%, and >50% residual tumor cells, respectively [26]. Patients with TRG1b were classified as responders, TRG2, and TRG3 as non-responders. Patients were treated with platinum/5-fluorouracil (5FU)-based chemotherapeutic regimes (Table 1). All surgical approaches included an abdominal D2-lymphadenectomy [27].

Follow-up data were obtained from the tumor data management of the University Hospital of Augsburg. Median follow-up was calculated by the inverse Kaplan–Meier method [28].

This study was approved by the Institutional Review Board at the Ludwig-Maximilians-University of Munich (reference: 20-0922) and was performed in accordance with the declaration of Helsinki.

### 2.2. Tissue Microarray Construction

All eligible histological sections were first re-evaluated using a light microscope to verify the diagnosis. Representative slides of each tumor were digitalized using a Pannoramic SCAN II scanner (3DHISTECH, Budapest, Hungary), and five areas, consisting of normal tissue (1×), central tumor (2×), and a tumor invasion front (2×) were selected. Based on the marked areas, formalin-fixed paraffin-embedded (FFPE) tumor samples were subsequently automatically assembled into a tissue microarray (TMA) using a TMA Grandmaster (3DHISTECH, Budapest, Hungary) with a core size of 1 mm.

### 2.3. Immunohistochemistry and In Situ Hybridization

**Figure 1:**
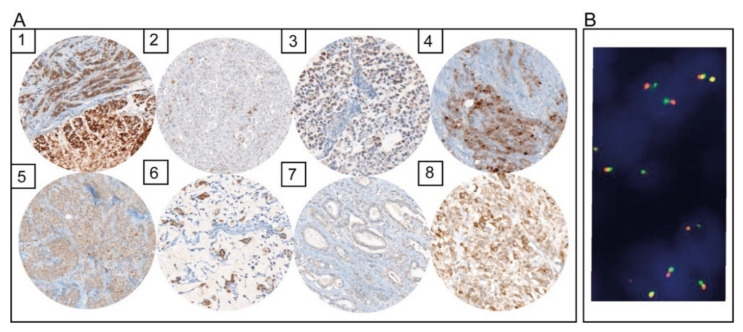
(**A**) Cases with immunohistochemical TRK expression (1–7) and a positive control of TRK immunohistochemistry (8); (**B**) FISH analysis of NTRK 1 of case 1 without the detection of break-apart signals.

**Figure 2:**
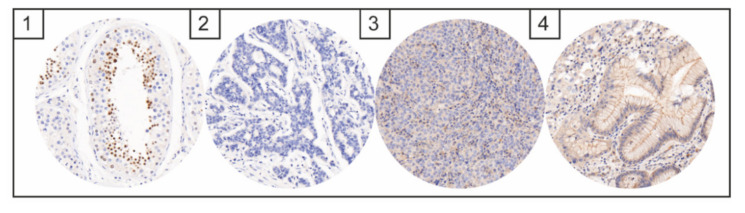
Images of immunohistochemical NUT expression: Spermatogenic cells as positive staining control (1); images of negative NUT staining (2–4); image with unspecific background staining in tumor cells (3); and normal foveolar epithelium (4).

Immunohistochemical staining was performed on 2 µm sections from each TMA using primary antibodies for TRK (RBT-TRK (ready-to-use (RTU)), Bio SB, Santa Barbara, CA, USA, BSB-2376), NUT (C52B1 (1:50), Cell Signaling Technology, Danvers, MA, USA, 3625), and ALK (D5F3 (RTU), Roche Diagnostics, Mannheim, Germany, 790-4794). For TRK, a Ventana BenchMark ULTRA platform with an iVIEW DAB detection system was used (Roche, Mannheim, Germany). Staining for NUT and ALK was performed on a BOND Rx platform with a BOND Polymer Refine Detection Kit (Leica Biosystems, Nussloch, Germany). Adequate controls were used for the quality control of the staining (Figure 1 and Figure 2).

The stained sections were digitalized, and evaluations were performed with a 3DHISTECH Caseviewer (3DHISTECH, Budapest, Hungary) by one pathologist (B.G.) and one trained researcher (M.G.), that independently reviewed the slides. Discrepant cases were discussed with a senior pathologist (B.M.), and a consensus was established. The investigators were blinded to both the clinicopathological data and outcome.

The immunohistochemical expression of NUT was evaluated as described by French et al. [23]. Only the staining of more than 50% of tumor cell nuclei was considered NUT positive [23]. According to Chon et al. [14], a case is considered ALK positive if at least 10% of the tumor cells showed moderate cytoplasmic, nuclear or membranous staining. The staining intensity was divided into weak (light-brown staining), moderate (medium-brown) and strong (dark-brown) [14]. Any cytoplasmic or nuclear staining with TRK was considered positive.

### 2.4. TCGA Classification

Tumors were classified in analogy to TCGA-classification [7] as proposed by Setia et al. and Ahn et al. [29,30] and, as described previously, in EBV^+^, mismatch repair deficient (MMRD), genomically stable (GS) and chromosomally instable (CIN) cases. Cases that showed nuclear staining by EBER-CISH were considered EBV positive. The presence of mismatch repair deficiency (MMRD) was stated in the case of loss of the nuclear expression of MSH6 or PMS2. GS cases were identified according to aberrant E-cadherin expression. E-cadherin was considered positive if membranous staining was present in more than 50% of tumor cells [31]. Tumors were classified as CIN if an aberrant p53 expression pattern was present. p53 expression was considered aberrant if tumor cells showed complete loss of nuclear expression or if they showed staining with strong intensity in more than 60%. The staining of less than 60% with weak to moderate intensity was considered a wild-type expression pattern [32,33]. Cases that did not meet the above criteria were designated as unclassifiable.

### 2.5. Next-Generation Sequencing (NGS) of NTRK-Positive Cases

RNA isolation and quantification: A 2 µm-thick section was obtained from the FFPE tissue and then stained with hematoxylin and eosin (H&E) for pathologic evaluation. Tumor cells were acquired using microdissection under histomorphological control. The percentage of tumor cells in the microdissected areas varied between 30% and 80% (mean 70%). RNA was isolated with a Maxwell^®^ 16 LEV RNA FFPE Purification Kit (Promega, Madison, WI, USA, AS1260) and fluorometrically quantified with QuantiFluor^®^ RNA System (Promega, Madison, WI, USA, E3310). For long-term storage, the samples were kept at −80 °C.

Library preparation and next-generation sequencing: RNA panel sequencing was performed for the molecular analysis of gene fusions. The library was prepared using AmpliSeq^TM^ Library PLUS for Illumina^®^, AmpliSeq^TM^ for Illumina^®^ Focus Panel RNA pool and AmpliSeq^TM^ CD Indexes for Illumina^®^. The RNA-input varied between 1 and 100 ng RNA/sample. The final cDNA libraries were fluorometrically quantified with QuantiFluor^®^ ONE dsDNA System (Promega, E4871), pooled to target a minimum of 100.000 reads for each tumor sample, diluted to a final concentration of 9 pM and sequenced by synthesis on the Illumina MiSeq using paired 150 bp reads on a V2 flow cell (Illumina, San Diego, CA, USA, MS-102-2002). BCL files were converted to FASTQ and fusion files using the RNA amplicon workflow application on the LRM software from Illumina. Seven cases with immunohistochemical TRK expression were analyzed by NGS.

### 2.6. Fluorescence In Situ Hybridization (FISH) Analysis of NTRK-Positive Cases

The analyses of the seven cases with immunohistochemical TRK expression were performed on 1 µm-thick whole-slide tissue sections of FFPE samples. For the detection of NTRK rearrangements, ZytoLight SPEC NTRK1 (Z-2167), NTRK2 (Z-2205), or NTRK3 (Z-2206) Dual Color Break Apart Probes (Zytovision, Bremerhaven, Germany) were used according to the manufacturer’s instructions.

## 3. Results

### NTRK, ALK, and NUT Expression

Seven cases (1.5%) with TRK expression were identified by immunohistochemistry (Figure 1A). Three cases (cases 1, 4, and 5) presented with moderate to strong cytoplasmic positivity in the majority of tumor cells. One case showed a moderately strong single-cell expression of TRK (case 2). One case showed very weak nuclear expression (case 7), and cases 3 and 6 presented moderately strong cytoplasmic expression in signet ring cells. In all seven cases, no fusion of the NTRK 1, 2, or 3 gene could be found by FISH (Figure 1B). In addition, next-generation sequencing revealed no NTRK fusion.

**Figure 3:**
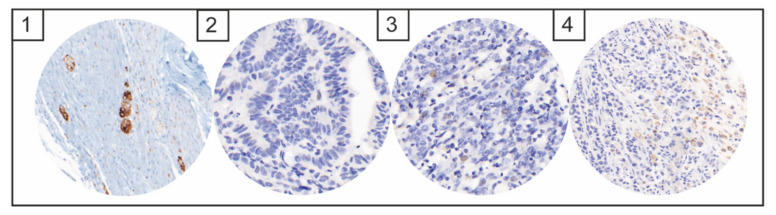
Images of immunohistochemical ALK expression: Ganglion cells of the appendix as a positive staining control (1), image of negative ALK staining (2), case with weak nuclear single-cell expression (3), and weak membranous unspecific staining pattern in signet ring cells (4).

No cases with immunohistochemical NUT expression could be detected (Figure 2). Only nonspecific cytoplasmic staining reaction was seen, with no specific nuclear staining reaction. No cases with positive ALK expression could be identified (Figure 3). Weak nuclear, membranous or cytoplasmic expression was seen in six cases but in less than 10% of the tumor cells in each case.

## 4. Discussion

Alterations of *ALK, NTRK*, and *NUT* were identified in low frequencies in various tumor entities. To the best of our knowledge, no prior study has evaluated the existence of ALK, TRK, and NUT in a large cohort of gastric carcinomas [13,22,24]. This study addressed this issue and analyzed the frequencies of ALK, TRK, and NUT alterations in gastric adenocarcinomas with or without neoadjuvant CTx.

*ALK* is a membrane-bound enzyme with tyrosine kinase activity that is physiologically present in the brain and peripheral nervous system only during the embryonic phase, which rapidly regresses after birth and is expressed in adults at low levels exclusively in the central nervous system (CNS). Detection outside the CNS is thus indicative of cell abnormality [34,35]. Gene fusions are strong tumor drivers and therapeutic targets amenable to tyrosine kinase inhibitors such as crizotinib [11,12,36]. Currently, the best studied *ALK* fusions are *NPM1-ALK* in anaplastic large-cell lymphoma (ALCL) and *EML4-ALK* in non-small cell lung cancer (NSCLC), which harbor *ALK* fusions in approximately 5% of cases [9,10].

*NUT* is typically expressed exclusively in post-meiotic spermatids. Through gene fusion, *NUT* leads to the extensive acetylation of chromatin which alters the transcription of oncogenes, blocks the differentiation of cells, and drives uncontrolled growth [23]. *NUT* is responsible for the development of the highly aggressive tumor entity of NUT carcinomas (NCs) that mainly affects young adults, is associated with a very poor prognosis and has been shown not to benefit from chemotherapy [23,24,37].

In our cohort of nearly 500 gastric adenocarcinomas, no cases with ALK or NUT expression could be detected. Alese et al. [13] detected one case with *ALK* fusion examining 25 signet-ring-cell adenocarcinomas of the stomach by FISH. Ying et al. [15] found two (0.44%) cases of ALK-positive colorectal carcinomas but no case with an ALK-positive gastric carcinoma in 182 cases. In our study, the analysis of *ALK* was performed only by immunohistochemistry and not using FISH. However, a recent study on lung carcinomas showed that immunohistochemistry is superior to FISH in the detection of altered *ALK* [36]. Dickson et al. described one case of gastric NUT-associated tumor [24]. Given the rarity of reports regarding *ALK* and *NUT* alterations in gastric cancer, our results indicate that neither *ALK* nor *NUT* fusion plays a relevant role in gastric cancer.

*NTRKs* encode three TRKs and influence the development and function of the central and peripheral nervous systems. Gene fusion can give rise to uncontrolled TRK activation and thus the uncontrolled activation of underlying signaling pathways, such as the MAPK-ERK or the PI3K. To date, two TRK inhibitors, namely larotrectinib and entrectinib, have been approved for the treatment of solid tumors with NTRK fusion. Others are currently being evaluated in clinical trials [17,18,19,20,21]. In 2020, Shinozaki-Ushiku et al. [22] identified the first case of gastric carcinoma in which altered immunohistochemical TRK expression and the associated gene fusion transcript could be detected by RNA-seq. However, prior to this, several studies had already demonstrated altered gene expression of TRK in gastric carcinomas [16,38,39]. For example, Lee et al. [40] detected TRK alteration in one of 66 gastric carcinomas by immunohistochemistry and FISH, but no *NTRK1* rearrangement was confirmed by NGS. We found seven cases with a immunohistochemical expression of panTRK. However, by analyzing these cases with NGS, no gene fusion transcript could be detected. As is the case for *ALK* and *NUT*, *NTRK* fusions do not seem to be relevant in gastric cancer. On the other hand, panTRK immunohistochemistry has had a relatively low false-positive rate and therefore seems to be an adequate method for screening cases with a specific indication.

Despite the comprehensive analysis of a large cohort, our study has limitations mainly related to its retrospective nature. Our study should be considered an exploratory analysis, and the results should be validated in independent prospective cohorts.

## 5. Conclusions

In summary, our results indicate that neither the expression of ALK, NUT, nor TRK plays a relevant role in gastric cancer. On the other hand, panTRK immunohistochemistry has had a relatively low false-positive rate and therefore seems to be an adequate method for screening cases with a specific indication.

## Figures and Tables

**Table 1:** Clinicopathological characteristics.

Variable		*n* = 477	%
Median age (range) (years)		70.0 (30.0–95.0)	
Median survival (range) (months)		58.0 (49.9–66.1)	
Sex	female	312	65%
	male	165	35%
T status	pT1/2	159	33%
	pT3/4	318	67%
N status	negative	178	37%
	positive	299	63%
Distant Metastasis	no	247	52%
	yes	197	41%
	NA	33	7%
Grading	low grade	162	34%
	high grade	304	64%
	NA	11	2%
Lymphovascular invasion	negative	287	60%
	positive	190	40%
Vascular invasion	negative	401	84%
	positive	76	16%
Lauren	intestinal	266	56%
	non-intestinal	211	44%
Localization	proximal (AEG II/III, Cardia)	124	26%
	non proximal (Fundus/Corpus/Antrum)	335	70%
	NA	18	4%
R status	R0	403	84%
	R1	54	11%
	Rx	20	4%
TCGA	EBV+	25	5%
	MSI	61	13%
	GS	110	23%
	CIN	151	32%
	no classification	130	27%
Death	no	227	48%
	death	250	52%
Preoperative CTx	no	347	73%
	yes	130	27%
TRG *(n = 130)*	1b	9	7%
	2	36	28%
	3	82	65%
CTx regimen	Cis/Ox + 5-FU or Cap	34	27%
	Ox + 5-FU + Doc	45	35%
	Cis + 5-FU + Epi	41	32%
	Ox + Epi + Cap	5	4%
	others	2	2%

TCGA: The Cancer Genome Atlas; EBV+: EBV positive; MSI: microsatellite instable; GS: genomically stable; CIN: chromosomally instable; Cis, cisplatin; Ox, oxaliplatin; 5-FU, 5-fluorouracil; Cap, capecitabine; Doc, docetaxel; Pac, paclitaxel; Epi, epirubicin; Others, combination of Cis/Ox with other agents or cross over between different treatment regimens.

## Data Availability

The datasets generated during the current work are available from the corresponding author upon reasonable request.
